# The Prognostic Role of Ezrin Immunoexpression in Osteosarcoma: A Meta-Analysis of Published Data

**DOI:** 10.1371/journal.pone.0064513

**Published:** 2013-06-21

**Authors:** Hongtao Li, Daliu Min, Hui Zhao, Zhiyu Wang, Weixiang Qi, Shuier Zheng, Lina Tang, Aina He, Yuanjue Sun, Yang Yao, Zan Shen

**Affiliations:** Department of Oncology, Affiliated Sixth People's Hospital, Shanghai Jiaotong University, Shanghai, People's Republic of China; Johns Hopkins University, United States of America

## Abstract

**Background:**

The significance of ezrin immunoexpression and prognosis for osteosarcoma is still controversial. The aim was to provide a meta-analysis for ezrin immunoexpression and prognostic features of osteosarcoma patients.

**Methods:**

A detailed search was made in MEDLINE, EMBASE and the Web of Knowledge for relevant original articles published in English; methodological quality of the included studies was also assessed. Two reviewers extracted data independently. Studies were pooled and summary hazard ratios (HRs) and odds ratio (ORs) with corresponding confidence intervals (CIs) were calculated.

**Results:**

Final analysis of 318 patients from 5 eligible studies was performed. Combined HR of ezrin immunohistochemical staining suggested that positive immunoexpression had an unfavorable impact on osteosarcoma patients' overall survival (n = 223 in 4 studies; HR = 4.79; 95% CI: 1.50–15.30; *P* = 0.008) but not on event-free survival (n = 202 in 3 studies; HR = 1.59; 95% CI: 0.61–4.15; *P* = 0. 0.342). Combined OR of ezrin immunohistochemical staining indicated that positive immunoexpression was associated with recurrence (n = 134 in 2 studies; OR = 3.79; 95% CI: 1.49–9.64; *P* = 0.005) but not with serum ALP level (n = 160 in 2 studies; OR = 2.16; 95% CI: 0.09–52.50; *P* = 0.637) and histological response to neoadjuvant chemotherapy(n = 260 in 4 studies; OR = 0.87; 95% CI: 0.37–2.03; *P* = 0.740).

**Conclusions:**

The results of this meta-analysis suggest that ezrin positive immunoexpression confers a higher risk of recurrence and a worse survival in osteosarcoma patients. Large prospective studies are needed to provide solid data to investigate the precise prognostic significance of ezrin.

## Introduction

Osteosarcoma is the most common bone cancer in children and young adults [1], [2]. In the past decades, the combination of multi-agent neoadjuvant chemotherapy has improved 5-year survival from 20% to about 70% in patients without metastasis [3], [4]. However, patients that present with metastatic disease or who relapse with disseminated tumors usually have a poor prognosis. To improve patient's prognosis, several attempts have been made to develop methods for predicting metastasis, based on tumor metastasis-associated genes and proteins [3], [5], [6].

Ezrin, also known as cytovillin or villin2, is a member of the ERM (Ezrin-Radixin-Moesin) family, which act as membrane organizers and linkers between plasma membrane and cytoskeleton [7]. It is involved in cell adhesion to the extracellular matrix and in cell–cell interactions, Rho mediated signal transduction pathway and the Akt mediated apoptotic pathway [8], [9]. These findings suggested that it might play a pivotal role in the development of human cancer.

The first research that identified an association between ezrin expression and outcome in osteosarcoma was published in 2004 [10]. In clinical research, a correlation between ezrin expression and malignancy has been reported among various human cancers, such as colorectal cancer, prostate cancer, ovarian cancer and melanoma [11]–[16]. We previously reported that mRNA level of ezrin could be a prognostic factor and a predictor of potential lung metastasis in Chinese osteosarcoma patients [17]. Recently, the discovery of small molecules that prevent osteosarcoma cells metastasis by directly targeting ezrin made it necessary to analyze the predictive value of ezrin in osteosarcoma with lines of published clinical investigations [17]–[23]. However, the prognostic value of ezrin expression in osteosarcoma patient's survival or other clinical features, such as recurrence and histological response to adjuvant chemotherapy, remains controversial. On the one side, several reports revealed that ezrin was a negative prognostic factor for survival for OS patients [17]–[19], [20], [23]; on the other side, several findings demonstrated that ezrin immunoexpression had no value of predicting the prognosis [18], [19].

In this study, we sought to conduct a systematic review and meta-analysis to estimate whether ezrin immunoexpression was associated with outcome of osteosarcoma patient. A pool of studies published between 2007 and 2010 on the association between ezrin immunoexpression and patient's survival, serum alkaline phosphatase (ALP) level, histological response to adjuvant chemotherapy and recurrence in osteosarcoma were analyzed. We found that ezrin mmunoexpression had an unfavorable impact on osteosarcoma patients' overall survival and was associated with recurrence.

## Methods

### Search Strategy and Selection Criteria

Medline, PubMed, Embase, and Web of Science were searched (last search was updated on 18^th^ Nov. 2012, using the search terms: ‘ezrin’, ‘cytovillin’, ‘villin2’, ‘vil2’,‘prognosis’ and ‘osteosarcoma’). All searched studies were retrieved, and their bibliographies were checked for other relevant publications. References of selected articles and reviews were also searched manually for additional relevant studies. When more than one of the same patient populations was included in several publications, only the most recent or complete study was used to avoid duplication of information.

Two independent reviewers assessed the eligibility of studies by reviewing titles and abstracts identified by the search. Differences were resolved by discussion. The inclusion criteria required that the identified articles were studies including: (1) ezrin expression evaluated in the primary osteosarcoma tissues; (2) relationship demonstrated between ezrin expression and osteosarcoma clinicopathological parameters or prognosis; (3) ezrin expression examined by immunohistochemistry; (4) articles published as a full paper in English; (5) studies provided sufficient information to estimate hazard ratio (HR) and 95% confidence interval (CI). The exclusion criteria were as follows:(1) duplicate studies on the same patients;(2) studies written in a language other than English;(3) letters, reviews, case reports, conference abstracts, editorials, expert opinion reviews and abstracts.

### Data Extraction and Study Assessment

Two investigators extracted data from eligible studies independently, discussed discrepancies, and reached consensus for all items. The following data were collected from each study: first author's name, year of publication, country, total number of patients, age, sex, and immunohistochemical technique, rate of ezrin positive expression. Duplication of data was avoided by matching the author's name and the name of the research centers.

### Statistical Analysis

Hazard ratio(HR) and its variance for each individual study were extracted or calculated based on the published researches according to the methods described by Parmar [24]. A HR>1 was considered as a risk factor for worse survival in patient with positive ezrin immunoexpression. This impact of ezrin on survival was considered as statistically significant if the corresponding 95% CI for the summary HR did not overlap 1 unit. Odds ratio (OR) was used to measure the relationship of ezrin immunoexpression and histological response to neoadjuvant chemotherapy, serum ALP level and recurrence. Heterogeneity between the studies was tested using Q-statistics. It was considered statistically significant if *P* value less than 0.10 and was also quantified using the *I* ^2^ metric (*I* ^2^<25%, no heterogeneity; *I* ^2^ = 25–50%, moderate heterogeneity; and *I* ^2^>50%, strong heterogeneity) [25], [26]. If the heterogeneity was existed, we used a random-effects model in place of a fixed-effects model [27].All *P* values were two sided. Statistical calculations were performed using STATA version 12.0. and Review manager 5.0.

## Results

### Study Selection and Characteristics

**Figure 1:**
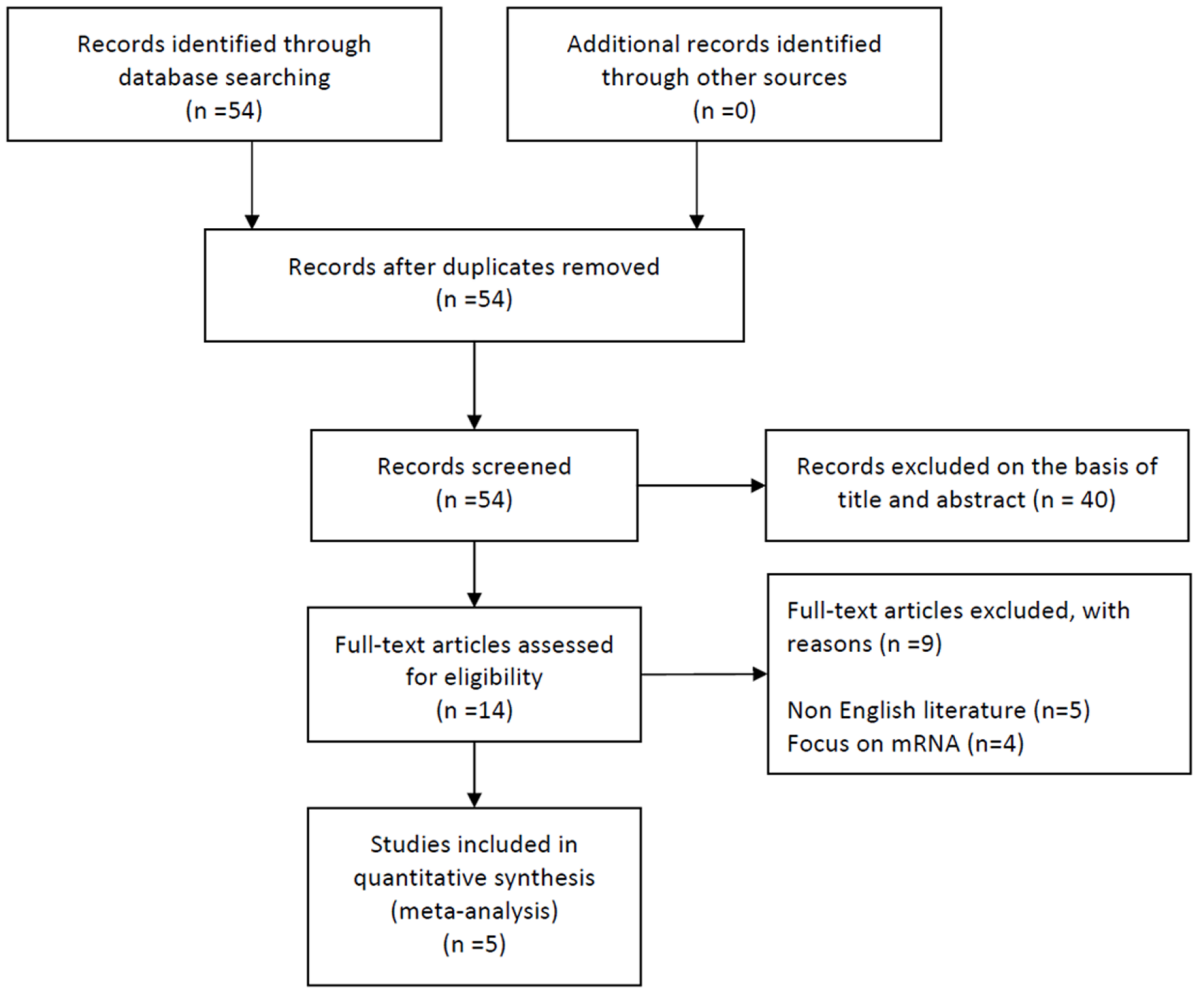
Flow diagram of study selection.

Fifty-five relevant citations were identified for initial review using search strategies as described previously. Of these, fifty were initially excluded after read the titles and abstracts (17 on cell lines; 20 meeting abstracts; 1 review article; 1 on tumor grades; 1 patent; 4 on mRNA expression; 5 published in Chinese) (Figure 1). Ultimately, the systematic literature search yielded a total of 5 studies comprising 318 patients for final analysis [18]–[21], [23]. The characteristics of retained 5 studies are listed in Table 1. These studies that were published from 2007 to 2010 met the inclusion criteria for our meta-analysis.

**Table 1:** Characteristics of eligible studies evaluating ezrin expression and OS or EFS in osteosarcoma patients.

Author (year)	Patient number (M/F)	Mean age (range)	Metastasis (yes/no)	Neoadjuvant chemotherapy protocol	Country	Antibody source	Dilution	Definition of positive	Ezrin positive (%) (cytoplasm/membrane)
Boldrini (2010)	52(27/25)*	15.9(7–25)**	28/24	BOTG2000/COG9754$	Brazil	LabVision	N/A	≥1 cells	76.5(50/50)
Kim (2009)	70(37/33)	15.7 (3.8–64.4)	0/70	N/A	Korea	NeoMarkers, Fremont, CA.	1∶300	>10% cells	55.7(N/A)
Ferrari (2008)	95(62/33)	16(4–39)	0/95	MTX, ADM, CDP,IFO#	Italy	Neomarkers, Fremont, CA.	1∶200	≥1% cells	80(49/51)
Salas (2007)	37(23/14)	15(6.7–54.2)	2/35	T10/SFOP OS94	France	Sigma	1/250	≥1% cells	62(N/A)
Kim (2007)	64(45/19)	19.4(4–58)**	0/64	Modified T10	Korea	Santa Cruz,CA	1∶100	≥1 cells	51.6(N/A)

N/A: not available;

* 34 samples were investigated;

** average age.

# MTX: methotrexate, ADM: doxorubicin, CDP: cisplatin, IFO: ifosfamide;

$ Brazilian Osteosarcoma Treatment Group 2000 and Children Oncology Group 9754.

The immunohistochemical technique for ezrin expression detection was summarized in Table 1. A HR on event-free survival (EFS) and/or overall survival (OS) could be extracted from these studies, respectively. Most of the survival data were obtained from five eligible studies [18]–[21], [23]. Four of the included studies investigated the association between ezrin immunoexpression and histological response to neoadjuvant chemotherapy response [19]–[21], [23], two studies analyzed the relationship between ezrin immunoexpression and serum ALP level [19], [20].

### Methodological quality of the studies

Four included studies were assessed by two authors with high levels of methodological quality (>6 stars) according to Newcastle–Ottawa quality assessment scale [28].

### Impact of ezrin immunoexpression on EFS and OS of osteosarcoma patients

**Figure 2:**
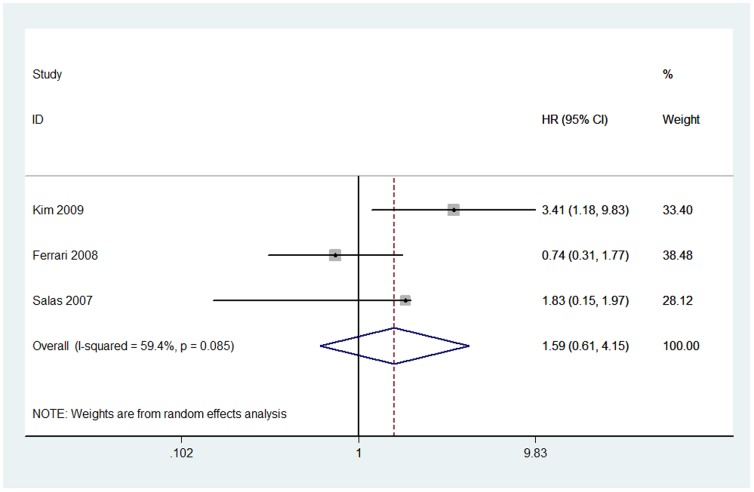
Forest plot showing the association between ezrin expression and event-free survival (EFS) of osteosarcoma. The summary HR and 95% CIs were shown (according to the random-effects estimations).

Three studies with a total of 202 osteosarcoma patients dealing with ezrin immunoexpression and EFS were meta-analyzed [19], [20], [23]. Because of heterogeneity (*I* ^2^ = 59.4%), a random effect model was adopted in this analysis. The pooled HR was 1.59 (95% CI: 0.61–4.15; *Z* = 0.95; *P* = 0.342), illustrating that ezrin immunoexpression was not significantly associated with the EFS of osteosarcoma patients (Figure 2).

**Figure 3:**
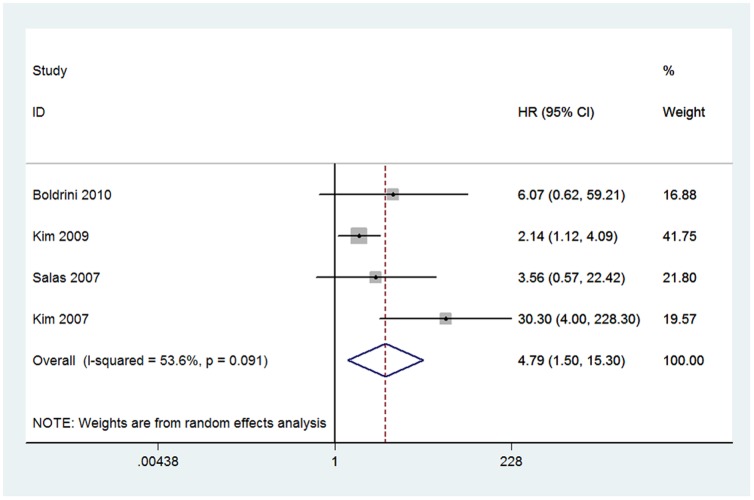
Forest plot showing the association between ezrin expression and overall survival (OS) of osteosarcoma. The summary HR and 95% CIs were shown (according to the random-effects estimations).

Four studies including 223 patients reported the correlation between ezrin immunoexpression and OS were also meta-analyzed [18], [20], [21], [23]. Due to heterogeneity (*I* ^2^ = 53.6%), a random effect model was selected. The combined HR was 4.79 (95% CI: 1.50–15.30; *Z* = 2.64; *P* = 0.008), which demonstrated that ezrin immunoexpression was significantly associated with the poor OS of osteosarcoma patients (Figure 3).

### Ezrin immunoexpression and histological response to neoadjuvant chemotherapy

**Figure 4:**
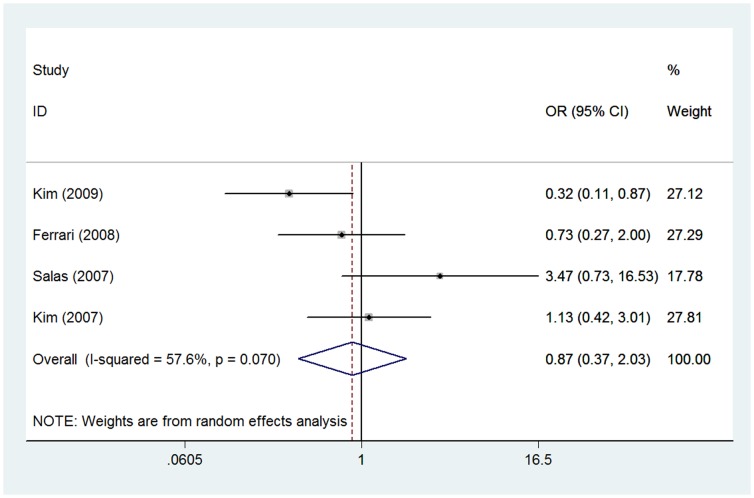
Forest plot showing the association between ezrin expression and histological response to neoadjuvant chemotherapy in osteosarcoma. The summary OR and 95% CIs were shown (according to the random-effects estimations).

Four of these included studies with 260 patients investigated the association between ezrin immunoexpression and histological response to neoadjuvant chemotherapy response [19]–[21], [23]. A random effect model was selected for analyze due to heterogeneity (*I* ^2^ = 57.6%). The combined OR was 0.87 (95% CI: 0.37–2.03; *Z* = 0.33; *P* = 0.740), suggesting that ezrin immunoexpression was not associated with histological response of osteosarcoma patients (Figure 4).

### Ezrin immunoexpression and serum ALP level

**Figure 5:**
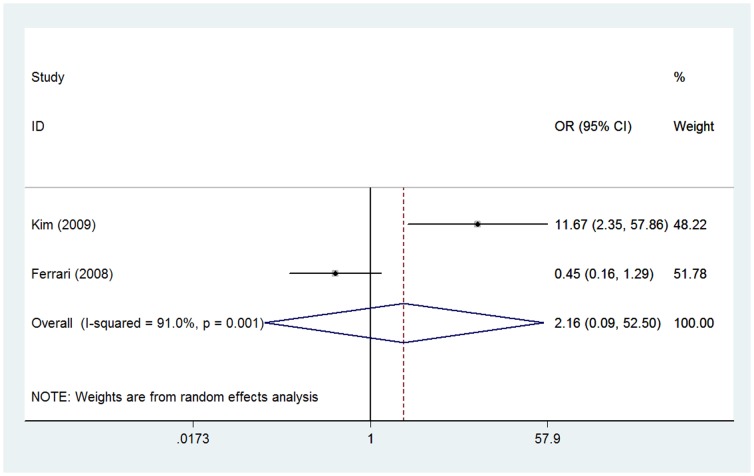
Forest plot showing the association between ezrin expression and serum ALP level in osteosarcoma. The summary OR and 95% CIs were shown (according to the random-effects estimations).

Two of these included studies with 160 patients investigated the association between ezrin immunoexpression and serum ALP level [19], [20]. Because of heterogeneity (*I* ^2^ =  91.0%), a random effect model was adopted in this analysis. The combined OR was 2.16 (95% CI: 0.09–52.50; *Z* = 0.47; *P* = 0.637), suggesting that ezrin immunoexpression was not correlated to serum ALP level (Figure 5).

### Ezrin immunoexpression and recurrence

**Figure 6:**
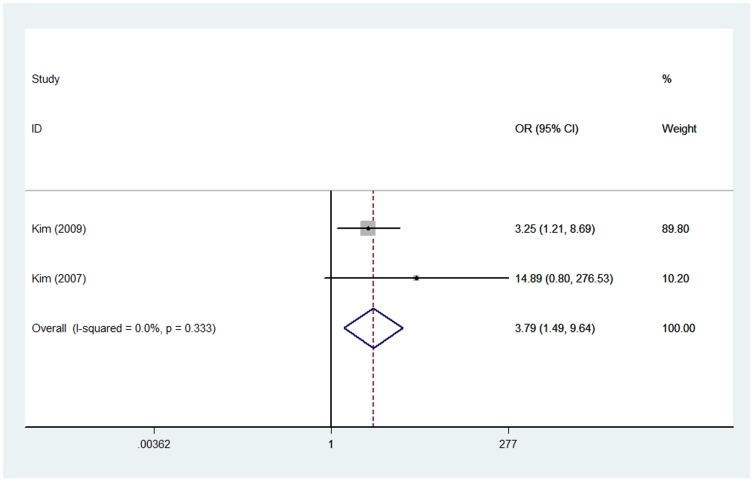
Forest plot showing the association between ezrin expression and recurrence in osteosarcoma. The summary OR and 95% CIs were shown (according to the fixed-effects estimations).

Two of these included studies with 134 patients investigated the relationship between ezrin immunoexpression and local recurrence [20], [21]. A fixed effect model was selected for analyze since no heterogeneity was detected (*I* ^2^ = 0.0%). The combined OR was 3.79 (95% CI: 1.49–9.64; *Z* = 2.80; *P* = 0.005), indicating that ezrin immunoexpression was significantly related to local recurrence (Figure 6).

### Assessment of Publication Bias

Because the number of study included in our meta-analysis was comparatively few, we did not draw funnel plot to demonstrate publication bias.

## Discussion

This meta-analysis showed that ezrin immunoexpression had an unfavorable impact on overall survival and was associated with recurrence in patients with osteosarcoma. No significant predictive associations were found between ezrin expression and EFS, histological response to neoadjuvant chemotherapy and ALP level.

However, these conclusions should be deciphered with several issues that could not be overcame: First, the results of ezrin immunoexpression were less powerful because only 5 studies were included for analysis with a relatively small sample size of 318 patients; second, as a result of small sample size, stratified analysis of different race was not performed. Because of the varied incidence by race at young population [29], we could not exclude race as a factor that might have influence on outcome of osteosarcoma patients.

In terms of survival, cautions should be paid to interpret the results of different significance of EFS and OS. First, four studies dealing with OS were included, whereas only three reports focus on EFS were eligible, and the recruiting patients were 233 and 202 respectively, which were relatively small sample size compare with other cancer studies. Second, there was 13.4% (30/223) of patients with metastasis were recruited in OS analysis, but it's only about 1% (2/202) of counterpart in EFS analysis. Since ezrin was demonstrated to be the key role of metastasis, it's obviously that different proportion of patients with potential to experience metastasis would make outcome different. These differences could lead to inhomogeneity of analysis.

It's reasonable to expect that recurrence rate accompanies with short EFS. However, the significant association between ezrin and recurrence did not result in a correlation between ezrin and EFS. This could be explained by different studies were included in recurrence and EFS (Figure 1) analysis (Figure 5).

Although ezrin was a key component factor for metastasis, it also played an important role in multidrug resistance and cell survival [30], [31]. Unfortunately, this meta-analysis showed no remarkable correlations between ezrin expression and histological response to neoadjuvant chemotherapy and ALP level despite the combined OR for ALP as high as 2.16. These negative results might due to that only two eligible studies with heterogeneity were analyzed. Therefore, more precise estimates of these relationships would be possible if more researches were included.

Meanwhile, some limitations in this meta-analysis should be noticed: First, marked heterogeneity of included studies existed in analysis except recurrence analysis. The heterogeneity was probably due to following reasons: primary antibodies from different companies, different cut-off points for positive staining, different immunoreactivity pattern (membrane/cytoplasm), baseline characteristics of patients (tumor stage, country) and the duration of follow-up. The variables mentioned above may make the pooled results less reliable. Second, there might be some biases if studies other than English were excluded. Third, the relatively small sample sizes in the included studies result in that even very powerful prognostic factors may not have become significant. Fourth, HRs were calculated from data or extrapolated from survival curves in the eligible studies, the HR information obtained by statistical software unavoidably developed a decrease of reliability.

## Conclusion

This meta-analysis suggests that ezrin was seems to be associated with a worse prognosis for OS and recurrence of patients with osteosarcoma with available evidence. However, more patients should be enrolled to improve statistical power and avoid clinical heterogeneity; moreover, large prospective studies are still needed to provide solid data to investigate the precise prognostic significance of ezrin.
